# Handbook of Gynæcological Operations

**Published:** 1887-12

**Authors:** 


*Handbook of Gynæcological Operations.* By ALBAN DORAN, F.R.C.S. Pp. 485. London: J. & A. CHURCHILL. 1887.

Although there is no definite statement to that effect, we must look upon this work as a more or less authoritative exposition of the practice of the Samaritan Free Hospital. In this sense it will be heartily welcomed by the profession. But, on the other hand, there is much in the work which is specially and peculiarly the author's own; and here also there is ground for congratulation in the issue of the work.

The work confines itself absolutely to the surgical or operative part of Gynæcology. This at once overlaps, and does not cover, the practice of the Samaritan. It overlaps it, in that an operation for extroversion of the bladder is described, which can scarcely be considered as gynæcological; and that operations on the kidney, which are performed in the Samaritan Hospital, are omitted. Most justly the book may be described as an exposition of Ovariotomy, full, accurate, and minute, with short accounts of such casual operations as happen to be attacked by men specially retained as ovariotomists.

While recognising the value, to one already trained in the ways of the operations described, of this admirable description of the work at the Samaritan, we doubt whether Mr. Doran has done himself or the operations justice. By a fuller and more intimate acquaintance with the work of others, the author would have written a book more worthy of general acceptance than this is, and more fully up to the level of his own capacity which is evidently of a high order. A perusal of the chapters on Hysterectomy, Perinæorraphy, and Ectopia vesicæ at once suggests this criticism; and reading and re-reading the admirable chapters on Ovariotomy confirms it.

Perhaps our slight disappointment with the work arises from our having expected too much. Still, it is a disappointment as regards degree of excellence, not as regards want of it. If, for some of the conditions, the mode of operating selected is not that which the majority of our best surgeons would adopt, in no case can the operation be described as other than good. And for the one central operation of the whole book, ovariotomy, we heartily express our belief that it gives the most complete written description in existence.

